# Tumor necrosis factor (TNF) induces astrogliosis, microgliosis and promotes survival of cortical neurons

**DOI:** 10.3934/Neuroscience.2021031

**Published:** 2021-11-16

**Authors:** Ebtesam M Abd-El-Basset, Muddanna Sakkattu Rao, Solaiman M Alshawaf, Hasan Kh Ashkanani, Abdulaziz H Kabli

**Affiliations:** 1 Department of Anatomy, Faculty of Medicine, Kuwait University, P.O. Box 24923, Safat 13100, Kuwait; 2 MD students, Faculty of Medicine, Kuwait University, Kuwait

**Keywords:** astrocytes, microglia, brain injury, TNF, cytokine

## Abstract

**Objectives:**

Neuro-inflammation occurs as a sequence of brain injury and is associated with production of cytokines. Cytokines can modulate the function and survival of neurons, microglia and astrocytes. The objective of this study is to examine the effect of TNF on the neurons, microglia and astrocytes in normal brain and stab wound brain injury.

**Methods:**

Normal BALB/c male mice (N) without any injury were subdivided into NA and NB groups. Another set mouse was subjected to stab wound brain injury (I) and were subdivided into IA and IB. NA and IA groups received intraperitoneal injections of TNF (1 µg/kg body weight/day) for nine days, whereas NB and IB groups received intraperitoneal injections of PBS. Animals were killed on 1^st^, 2^nd^, 3^rd^, 7^th^, and 9^th^ day. Frozen brain sections through the injury site in IA and IB or corresponding region in NA and NB groups were stained for neurodegeneration, immunostained for astrocytes, microglia and neurons. Western blotting for GFAP and ELISA for BDNF were done from the tissues collected from all groups.

**Results:**

The number of degenerating neurons significantly decreased in TNF treated groups. There was a significant increase in the number of astrocytes and microglia in TNF treated groups compared to PBS treated groups. In addition, it was found that TNF stimulated the expression of GFAP and BDNF in NA and IA groups.

**Conclusions:**

TNF induces astrogliosis and microgliosis in normal and injured brain and promotes the survival of cortical neurons in stab wound brain injury, may be by upregulating the BDNF level.

## 1. Introduction

Tumor necrosis factor (TNF) is a proinflammatory cytokine that regulates macrophage activity and involved in production of other pro-inflammatory cytokines, in addition to increasing prostaglandins and platelet activating factor. This suggests TNF's central role in the development of inflammatory diseases [1]–[3]. TNF has also been linked with resolution of inflammation through LPS-mediated efferocytosis of neutrophils by macrophages mediated via TNF [4]. As such, many inflammatory diseases have used agents targeting TNF in treatment regiments, including rheumatoid arthritis, inflammatory bowel disease, psoriatic arthritis, ankylosing spondylitis, juvenile chronic arthritis, atherosclerosis, and sepsis [5]–[8]. In a clinical trial of arthritis induced neuropathic pain, anti TNF therapy showed success in relieving neuropathic pain. However, patients had a higher risk of developing adverse neurological diseases such as multiple sclerosis, optic neuritis, distal acquired demyelinating symmetric neuropathy [9]–[15].

TNF is expressed by many cells, including astrocytes and microglial cells of the brain. TNF has two types of receptors (TNFR1 and TNFR2) which are made up of four cysteine repeats. Both receptors are expressed in various tissues, with TNFR2 being more specific to the cells of the immune system [16],[17]. The damaged brain contains high levels of TNF due to being released from microglia and astrocytes, after activation by pathogens or damage via pattern recognition receptors such as Toll-like receptors (TLRs) [18]–[20]. TNF has many roles in acute and chronic processes involving innate immunity, inflammation, infection, and pathogenesis of systemic and non-cerebral diseases [21].

Several studies have shown that TNF has a prominent role in the nervous system, however, these studies provide paradoxical roles of TNF, with some displaying a neuro-modulatory and protective effect in the normal or uninflamed brain. While others show a neurotoxic effect in the diseased or inflamed brain [22]. In the healthy CNS, TNF shows an important role in synaptic plasticity and modulating responses to neural injury [23],[24] and regulating synaptic gliotransmission of astrocytes [25],[26] as well as hippocampal homeostatic synaptic plasticity [27].

On the other hand, in pathological conditions, TNF is involved in the neuroinflammatory responses associated with various neurological disorders through de novo production of TNF by activated astrocytes and microglia [26],[28]. Astrocytes and microglia are two main glial cells involved in neuroinflammation and progression of neurodegenerative disease [29]. Astrocyte are the most abundant glial sub type in the CNS [30] and their activation is associated with secretion of proinflammatory cytokines such as IL-1β and TNF [31],[32]. Activated astrocytes have neuroprotective properties during inflammation through release of neurotrophic factors and maintaining the structural integrity of the blood brain barrier [33]. In addition, astrocytes have also shown the ability to interact and exert their control on microglia [34].

Research on hippocampal damage showed that increased TNF immunoreactivity in the hippocampus is associated with lower rates of survival in the early periods [35] suggesting the importance of further assessing crucial role of TNF in therapeutic strategies despite its known neurotoxic effects in various disease processes. The raising number of traumatic brain injuries (TBI) worldwide has led to an increased interest in research and awareness around the subject [36]. Understanding the mechanisms involved in the process of TBI, including inflammatory cytokines such as interleukin and TNF, helps us in predicting the prognosis, developing therapeutic strategies, as well as limiting the disability and mortality of the disease [37]. It is important to study the effects of TNF on the brain to better understand its role in pathogenesis and in treatment of certain diseases involving the brain.

Thus, TNF released during inflammatory process or infused from external source has effects on neurons, neurotrophic factors, microglia and astrocytes in and around the injury site. In spared nerve injury (SNI) of the sciatic nerve, tumor necrosis factor is shown to be released and upregulated in bilateral hippocampus and ipsilateral spinal dorsal horn with significantly reduced dendrite lengths and spine densities in the hippocampus and increased in spinal cord neurokinin-1 positive neurons [38]. However, brain-derived neurotrophic factor (BDNF) is decreased in the hippocampus but increased in the ipsilateral spinal dorsal horn after SNI. These alternations were associated with microgliosis in both spinal dorsal horn and hippocampus and mediated through TNF receptor-1 [38]. Treatment with TNF promote gliosis and inflammatory response of activated Müller cells, thus aggravating retinal ganglion cells injury in glaucoma [39]. Large amount of TNF is released from microglia during inflammation stimulates astrogliois. The elevations in TNF after neural injury precedes or occurs concomitantly with, the rise in glial fibrillary acidic protein mRNA, an early manifestation of astrogliosis [26]. A recent study showed the neuroprotective role of TNF-pretreatment in survival of human neural progenitor cells (hNPCs) in hypoxic-ischemic brain injury model [40].

Although the pro-inflammatory cytokine TNF is thought to be one of the major mediators of neuroinflammation, its role in brain injury, effects on neurons, microglia, astrocytes and trophic factors in and around the injury site remains ill-defined. TNF activity is very acute. It has been shown that TNF mRNA increases within 1 h in the ischemic injury core, and the expression of its immunoreactive protein increases within 2–6 h after the onset of ischemia in experimental models [41]. TNF increases in the serum of stroke patients as early as 6–12 h after symptom onset [38]. A recent study characterized the effect of acute exposure to low doses of TNF in a mouse hippocampal neuronal cell line (HT-22) and mouse primary cortical neurons. A rapid and significant mitochondrial dysfunction was observed after as little as 1.5 h of exposure to TNF which preceded cell death. This rapid action of TNF may be due to impairment of neuronal mitochondrial function [42]. In middle cerebral artery occlusion (MCAO), an experimental focal cerebral ischemia model, inhibition of microRNA-210 (miR-210) suppressed proinflammatory response and reduced brain damage in the acute phase of ischemic stroke. Further inhibition of miR-210 significantly reduced the expression of pro-inflammatory cytokines (TNF-α, IL-1β, and IL-6) and chemokines (CCL2 and CCL3) but had no significant effect on anti-inflammatory factors (TGF-β and IL-10).

As stated above, TNF has both neuroprotective and neurodegenerative roles. It is well known that activated glial cells release inflammatory factors (including TNF) by themselves and damage neurons. What is not known fully is that weather extraneously infused TNF will provide neuroprotection or not in stab-wound injury. Our hypothesis is that extraneously infused TNF will have neuroprotection in stab-wound injury. It will stimulate astrocytes and microglia to secrete neurotrophic factor, which in turn protect the neurons from degeneration. Our hypothesis is based on a few literatures which indicated protective role of intrinsic TNF which was released from activated astroglia and microglia [39] and extraneous TNF on hNPCs [40]. Accordingly, the objective of this study is to examine the effect of TNF on the neurons, microglia and astrocytes in normal brain and stab wound brain injury in the cerebral cortical region.

## 2. Materials and methods

### 2.1. Animals

Two months old BALB/c male mice were used in the present study. Mice were maintained in the Animal Resources Centre, Faculty of Medicine, Kuwait University. Mice were fed with food and water ad-libitum. Animals were maintained in 12:12 dark: light cycle and room temperature were maintained at 25 ± 2 °C. The animal surgery, treatment protocol and maintenance were according to the approved protocol of the Institutional Animal Care and Use Committee of Kuwait University, which follows the recommendations of NIH Guidelines and the Guide for the Care and Use of Laboratory animals. A minimum and sufficient number of animals were used due to good experimental design and planning. Experiment that involved brain injury were performed on the animals under anesthesia, aseptic technique, and with the least harm inflicted to the animals. Ketamine (40 mg/kg)-xylazine (5 mg/kg) cocktail (ketamine hydrochloride/xylazine hydrochloride solution, Catalogue number: K4138, Sigma-Aldrich Inc., St. Louis, MO, USA) was used for anesthetizing the mice during stab-wound injury. Antiseptic betadine solution was applied on the skin wound after stab wound injury. All experimental protocols were approved by the institutional ethical committee as stated above.

### 2.2. Experimental animal groups, stab wound injury and treatment

This experiment was designed to investigate the role of TNF treatment on astrocytes and microglia, and neuronal survival in stab wound model of brain injury (an in vivo model of penetrating brain injury) and normal brain. A total of 264 BALB/c male mice were divided into two sets (n = 132/set). The first set of mice (N, normal mice) were not subjected to any injury and were subdivided into A (for TNF treatment, NA, n = 66) and B (for PBS treatment, NB, n = 66) groups. The second set mice were subjected to stab wound brain injury (I, injured mice). A stab wound was done on the cerebral cortex of these mice as described earlier [38]. Briefly, mice were anaesthetized with ketamine (40 mg/kg)-xylazine (5 mg/kg) mixture (Sigma Chemicals, St. Louis, USA), a stab-wound (penetrating brain injury) was made by inserting 21-gauge sterile needle into right frontal cerebral cortex following mouse brain atlas [43] [anteroposterior (AP) = 1.8 mm (in front of bregma), dorsoventral (DV) = 2 mm (from skull surface) and Lateral (L) = 3 mm (right side from midline)]. These injured mice were subdivided into A (TNF treatment, IA, n = 66) and B (PBS treatment, IB, n = 66) groups. NA and IA groups received intraperitoneal injections of TNF (1 µg/kg body wait/day) for nine days. First dose was given 1 hr after the stab-wound injury [44]. NB and IB groups received intraperitoneal injections of PBS (0.5 mL) for nine days, at a time matching with that of TNF treatment in NA and IA groups. Since TNF was dissolved in PBS, we used PBS as vehicle control. Animals were killed on 1^st^, 3^rd^ and 7^th^ days after the beginning of the experiment (n = 18 for each time point, in all groups: six for morphological study and six for western blot analysis of GFAP and six for ELISA analysis of BDNF). For BDNF analysis additional two time points 2^nd^ and 9^th^ day were used (n = 6 for each time point, in all groups). Frozen brain sections through the injury site in IA and IB or corresponding region in NA and NB groups were stained for Fluoro-Jade B (neurodegeneration), Immunostained of for GFAP (astrocyte marker), Iba-1 (microglia), and NeuN (neuronal marker). Morphological alterations are not as rapid as biochemical changes. Hence, we did morphological studies on 1^st^, 3^rd^ and 7^th^ day, excluding 2^nd^ and 9^th^ day time points. Western blotting (for GFAP) and ELISA (for BDNF) were done from the tissues collected from the injury sites in IA and IB groups and corresponding site in NA and NB groups. BDNF analysis was done on the tissue collected on 1^st^, 2^nd^ 3^rd^, 7^th^ and 9^th^ day (i.e. additional 2^nd^ and 9^th^ day groups for BDNF analysis). We wished to see BDNF level on 2^nd^ day because alterations in levels of BDNF is quicker than morphological changes in the early post lesion days. We included 9^th^ day in order to see the additional effect during two extended days (7^th^ to 9^th^).

Nnumber of mice used in different groups for morphological and biochemical studies were shown in table 1. A total of 264 mice were used in the present study. Mice under morphological studies (N = 72) were equally distributed for each group for each time point of study (6 mice/group/time point of study) for Fluoro-jade B staining, Immunostaining for Astrocytes (GFAP), microglia (Iba1) and neurons (NeuN). Mice under biochemical studies (N = 192) were distributed 12 mice/group for 1^st^, 3^rd^ and 7^th^ day for analysis of BDNF by ELISA method (n = 6/group) and analysis of GFAP by western blot (n = 6/group), and 6 mice/group for 2^nd^ and 9^th^ day (for analysis of BDNF only by ELISA method (n = 6/group).

**Table 1.** Number of mice used for morphological and biochemical studies.

Groups	Morphological study-(FJB Staining, immunostaining for GFAP, Iba1, NeuN)			Biochemical studies-(ELISA for BDNF and Western Blot for GFAP)					Total
	Day			Day					
	1^st^	3^nd^	7^th^	1^st^	2^nd^	3^rd^	7^th^	9^th^	
IA	6	6	6	12	6	12	12	6	66
IB	6	6	6	12	6	12	12	6	66
NA	6	6	6	12	6	12	12	6	66
NB	6	6	6	12	6	12	12	6	66
Total	24	24	24	48	24	48	48	24	264

### 2.3. Tissue fixation and sectioning

The animals were perfused transcardially with saline followed by 4% paraformaldehyde (Catalogue no. 158127, Sigma-Aldrich Inc., St. Louis, MO, USA) and their brains were dissected and fixed in 4% paraformaldehyde for 48 hours. Then brains were cryoprotected in 10%, 20%, and 30% sucrose solution as described before [45]. Thirty-micron, thick serial, coronal frozen sections were cut and collected in 24 well culture plates filled with phosphate buffer.

### 2.4. Fluoro Jade B (FJ-B) staining

To visualize degenerating neurons, the brain sections from non-injured and injured brains were processed for FJ-B histochemical staining. Fluoro-Jade B and C are an anionic fluorescein derivative useful for the histological staining of neurons undergoing degeneration. Fluoro-Jade B has a greater specific affinity for degenerating neurons than Fluoro-Jade B. We used FJ-B for staining the degenerating neurons around injury site.

The brain sections were mounted on gelatin coated slides and air dried at room temperature overnight. Slides were rehydrated and treated with 0.06% potassium permanganate followed by washing in distilled water. The sections were then treated with 0.004% FJ-B (Catalogue no. MFCD04974901, Fluoro-Jade B, Histo-Chem Inc., Jefferson, Arkansas, 72079, USA) in 0.1% acetic acid for 30 minutes with slow shaking in dark. The sections were rinsed in distilled water, dehydrated and mounted with DPX. Number of FJ-B stained degenerating neurons were counted in six randomly selected photograph/section taken around the injury site (n = 6/group in each time point) (Photographs were taken with 40x objective in an olympus fluorescence microscope). Finally, the number of degenerating neurons/mm^3^ tissue were calculated for each mouse as detailed below.

### 2.5. Immunostaining for astrocytes (GFAP), microglia (Iba-1) and neurons (NeuN)

The brain sections were treated with 3% hydrogen peroxide, then with 5% normal goat serum for 30 minutes. The sections were incubated with polyclonal rabbit anti-GFAP (1:100, Catalogue no. ab7260, Abcam Inc., Cambridge, United Kingdom, CB2 0AX), polyclonal rabbit anti-Iba-1 (1:500, Catalogue no. ab153696, Abcam Inc., Cambridge, United Kingdom, CB2 0AX) or mouse monoclonal anti-NeuN (1:1000, Catalogue no. ab104224, Abcam Inc., Cambridge, United Kingdom, CB2 0AX) antibody overnight at 4 °C. Glial fibrillary acidic protein (GFAP) is an intermediate filament protein of 52 kD found in glial cells such as astrocytes. We used anti-GFAP antibody for immunostaining astrocytes for identification of astrocytes around the injury site and for immunoblotting for GFAP in western blot analysis of GFAP content in the tissue around the injury site. Ionized calcium binding adaptor molecule 1 (IBA1), is a marker of microglia/macrophages. It is a 17-kDa protein that is specifically expressed in macrophages/microglia and is upregulated during the activation of these cells. Iba1 expression is up-regulated in microglia following nerve injury, central nervous system ischemia, and several other brain diseases. We used anti-Iba1 antibody for immunostaining astrocytes for identification of activated microglia around the injury site. NeuN (neuronal nuclei) antibodies are fundamental tools for staining mature neurons and studying neuronal development and differentiation. We used anti-NeuN antibody to immunostaining to identify the mature neurons around the injury site.

The sections were then incubated with biotinylated goat anti-rabbit IgG (1:200, Catalogue no. BA-1000, Vector Labs, Burlingame, CA, USA) or biotinylated horse anti mouse IgG (1:200, Catalogue no. BA-2000, Vector Labs, Burlingame, CA, USA) for 1 hour at room temperature. All sections were treated with ExtrAvidin peroxidase (1:20, Catalogue no. E2886, Sigma-Aldrich Inc., St. Louis, MO, USA) for 1 hour. The color was developed using DAB solution (Catalogue no. SK-4100, Vector Labs, Burlingame, CA, USA). Then the sections were dehydrated in ascending grades of ethyl alcohol, cleared in xylene and mounted with DPX.

### 2.6. Quantification of degenerating neurons and immunostained neurons, astrocytes and microglia

Number of degenerating neurons, immunostained neurons, astrocytes and microglia were quantified as described earlier [45]. Six photomicrographs were taken from each sections around the lesion site in stab-wound injury site in stab-wound injured mice or corresponding region in non-injury mice (Photographs were taken with 40x objective). Number of Fluoro-Jade B stained degenerating neurons, NeuN positive neurons, GFAP stained astrocytes and Iba-1 stained microglia were counted in these six randomly selected field photographs. From each mouse six sections (60 µm apart) were selected for quantification. Finally, number of FJ-B stained degenerating neurons, NeuN positive neurons, astrocytes or microglia/mm^3^ tissue were calculated for each mouse as described earlier [45]. Briefly, total number of degenerating neurons, neurons, astrocytes or microglia/mm^3^ tissue were quantified by using the data collected from a total of 36 sections/mice (n = 6/group) using the formula: T = (N × V)/t, where N is the numerical cell density, V is the volume of the lesioned tissue around the lesion site used for quantification and t is section thickness (30 µm). The numerical cell density (N) was calculated by using the area of the section used for quantification, area of each field used for quantification and total cell counts in six randomly selected fields. NIS-Elements software (NIS-Elements-D2.20) was used for area measurement. The volume (V) was calculated by multiplying the area of section with section thickness (30 µm), inter-section distance (60 µm) and number of sections used (6 sections).

### 2.7. Polyacrylamide gel electrophoresis (PAGE) and immunoblotting

Animals were perfused with 50 mL of cold saline. Tissue around the injury site (5 mm^3^) and from corresponding regions of non-injury brains were removed and snap frozen in liquid nitrogen and stored at –80 °C until Western Blot analysis as described earlier [46]. Briefly, tissue was thawed and incubated in ice cold radioimmunoprecipitation assay (RIPA) lysis buffer [Catalogue no. sc-24948, Santa Cruz Biotechnology, Inc., Dallas, Texas 75220, USA; with sodium orthovanadate (0.5 mM), and the protease inhibitors, phenylmethanesulfonyl fluoride (PMSF; 1 mM), aprotinin (10 µg/ml), leupeptin (1 µg/ml)] for 10 minutes. Tissue was homogenized in cold, in a tissue homogenizer for 3–5 minutes, homogenate was centrifuged at 14,000 rpm at 4 ºC for 5 min to collect the supernatant. The protein concentration in the samples was determined using a spectrophotometer. All samples (75 µg protein/well) were analyzed electrophoretically on a 10% SDS-PAGE gel [46]. The proteins in the gel were transferred to nitrocellulose membrane [47]. After transfer, the membranes were incubated for 1 h with 5% skim milk in Tris-buffered saline-Tween 20 (TBST). The immunoblots were probed with rabbit anti-GFAP (Catalogue no. ab7260, Abcam Inc., Cambridge, United Kingdom, CB2 0AX) diluted in 5% skimmed milk in TBST, and rabbit anti-Glyceradehde-3-phosphate dehydrogenase (GAPDH, Catalogue no. SAB1410512, Sigma Aldrich Inc., St. Louis, MO, USA) were used as endogenous sample loading control. This was then followed by incubation with affinity-purified goat anti-rabbit conjugated to horse-radish peroxidase (1:100, Catalogue no. 401353, Sigma-Aldrich, St. Louis, MO, USA). Immunoreactive bands were visualized using enhanced chemiluminescence system (ECL, Catalogue no. sc-2048, Santa Cruz Biotechnology, Inc., Dallas, Texas, USA). The difference in the band intensities on exposed films were determined by densitometric scanning. Intensity of bands were quantified in the Image-J image analysis software.

### 2.8. ELISA for BDNF

Tissue samples (5 mm^3^ tissue around injury site) were collected snap frozen in liquid nitrogen, stored at (–80 °C) and were weighed immediately to get the wet weight of the samples and analyzed for BDNF content as described earlier [45]. Brain-derived neurotrophic factor is a protein (Molecular Weight of mature BDNF: 14 kDa). In humans, it is encoded by the BDNF gene. BDNF is a member of the neurotrophin family of growth factors, which are related to the canonical nerve growth factor. We used the ChemiKine Brain Derived Neurotrophic Factor, Sandwich ELISA kit for analysis of BDNF content in the tissue around the injury site. ELISA plates pre-coated with anti-BDNF antibodies were used for analysis of BDNF in the tissue. Each sample was transferred to 250 µL of ice-cold homogenization buffer and homogenized for one minute in a tissue homogenizer. Composition of the homogenization buffer was 100 mM Tris/HCl, pH 7, 2% bovine serum albumin (BSA), 1 M NaCl, 4 mM EDTA.Na_2_, 2% Triton X-100, 0.1% sodium azide and the protease inhibitors (5 µg/mL aprotinin, 0.5 µg/mL antipain, 157 µg/mL benzamidine, 0.1 µg/mL pepstatin A and 17 µg/mL phenylmethyl-sulphonyl fluoride). The lysate from each sample was centrifuged at 14,000 revolutions/minute (rpm) for 30 min at 4 °C and the supernatant solutions were collected. The supernatant from each sample was frozen for subsequent measurements of BDNF by using ChemiKine brain derived neurotrophic factor (BDNF) sandwich ELISA kit (Catalogue no. CYT306, Merck Millipore, Billerica MA, USA) following the protocol provided in the kit. Briefly 100 µL of standards or appropriately diluted samples were added into each flat bottom wells, pre-coated with mouse anti-Human BDNF monoclonal antibody and incubated at 4 °C overnight on a shaker. Wells were washed three times with 250 µL of diluted wash buffer. 100 µL of the diluted biotinylated mouse anti-BDNF monoclonal antibody (1:1000 in sample diluent) was added to each well and incubated at room temperature for three hours on a shaker. Wells were washed again three times with 250 µL of diluted wash buffer. 100 µL of the diluted streptavidin-HRP conjugate solution (1:1000, in sample diluent) was added to each well and incubated at room temperature for one hour on a shaker. Wells were washed three times with 250 µL of diluted wash buffer. 100 µL of TMB substrate (3, 3′, 5, 5′-tetramethylbenzidine) was added to each well and incubated at room temperature for 15 minutes. Reaction was stopped by adding 100 µL of stop solution to each well. The wells were read immediately in an ELISA plate reader at 450 nm. Optical density of standard solution was plotted against known concentration of the standards to get the standard curve. Unknown concentration of the BDNF in the samples were calculated by plotting their OD values into the standard curve.

### 2.9. Statistical analysis

Number FJ-B positive degenerating neurons, NeuN stained normal neurons, number of GFAP stained astrocytes, Iba-1 stained microglia, were counted in six randomly selected fields in each section around the injury sites and corresponding area from non-injury brains. From each mouse six sections (60 µm apart) were selected for quantification. Six mice were used in each group. Finally, mean number of astrocytes or microglia or degenerating neurons or neurons/mm^3^ tissue were calculated as described above. The data were expressed as Mean ± SEM and were analyzed by two-tailed Student's t-test or Two-way ANOVA, followed by Bonferroni's post-test. p-values < 0.05 was considered statistically significant.

## 3. Results

In all mice with stab-wound injury, tip of the needle remained in the frontal cortical region. It did not injure any other nuclei or structures (striatum, thalamic nuclei or hippocampus).

### 3.1. Effect of TNF on neurons around the injury site

**Figure 1.**
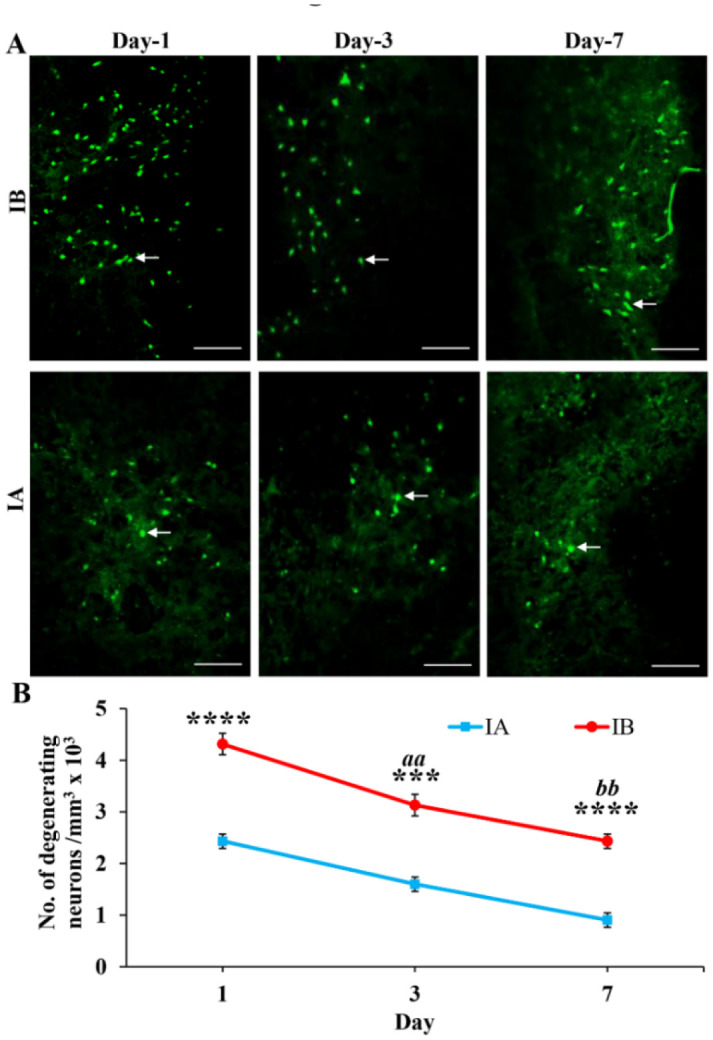
(A) Photomicrographs showing Fluro Jade B stained degenerating neurons (arrows) around the stab-wound injury site in injured mice treated with TNF (IA) and injured mice treated with PBS (IB) on 1^st^, 3^rd^ and 7^th^ day. As there are no degenerating neurons stained by Flurojade-B in non-injured mice brain sections [both treated with TNF (NA) and PBS (NB)], those photographs were not included. Note the decreased distribution of the degenerating neurons in TNF treated mice (IA) compared to PBS treated mice (IB) at all-time points. Scale bar = 60 µm. (B) Graph showing the number of degenerating neurons on 1^st^, 3^rd^ and 7^th^ day in injured mice treated with TNF (IA, n = 6 for each time point) and injured mice treated with PBS (IB, n = 6 for each time point). Note the significantly decreased number of degenerating neurons in TNF treated mice compared to PBS treated mice on 1^st^ day (Student's t-test, **** p < 0.0001), 3^rd^ day (*** p < 0.001) and 7^th^ day (**** p < 0.0001). Also note that in both IA and IB degenerating neurons progressively decreased from 1^st^ day to 3^rd^ day (^aa^ p < 0.01) and 3^rd^ day to 7^th^ day (^bb^ p < 0.01). Since there were no FJ-B stained neurons in non-injured groups (NA and NB), there is no quantified data shown in the graph.

**Figure 2.**
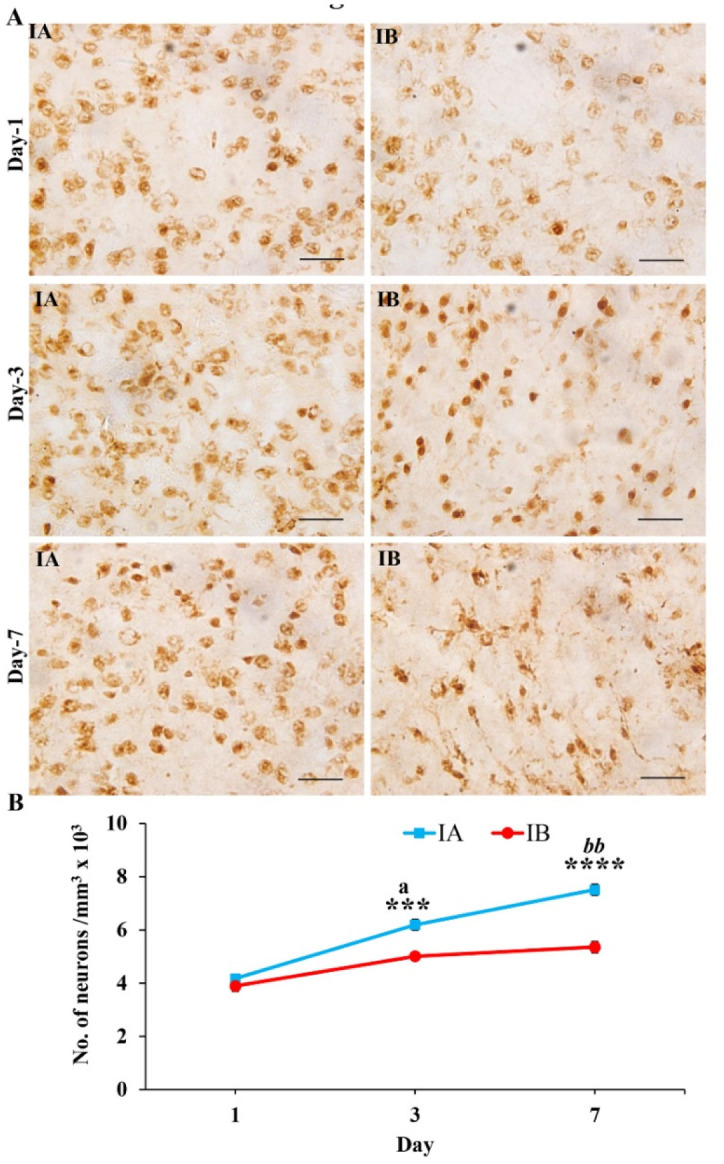
(A) Photomicrographs of the brain section from stab wound injured mice showing NeuN immunopositive neurons in TNF treated mice (IA) and PBS treated mice (IB) on 1^st^, 3^rd^ and 7^th^ day. Note the presence of many neurons in the TNF treated mice (IA, indicating less neuronal degeneration) compared to PBS treated mice (IB) at all-time points. Scale bar = 50 µm. (B) Graph showing the number of NeuN immunopositive neurons in TNF (IA, n = 6 for each time point) and PBS (IB, n = 6 for each time point) treated mice on 1^st^, 3^rd^ and 7^th^ day. Note significantly more number of neurons in TNF treated mice compared to PBS treated mice at 3^rd^ (Student's t-test, *** p < 0.001) and 7^th^ day (**** p < 0.0001). Also note that in IA group number of neurons progressively increased from 1^st^ day to 3^rd^ day (^a^ p < 0.05) and 3^rd^ day to 7^th^ day (^bb^ p < 0.01), but not in IB group. Since there was no difference in the number of NeuN positive neurons in non-injured mice treated with TNF (NA), and treated with PBS (NB), those photographs and data were not shown.

Flouro Jade B (FJ-B) staining revealed degenerating neurons around the injury site in the stab wound brain injury. Distribution of degenerating neurons in injured mice treated with TNF was less dense compared to injured mice treated with PBS at 1^st^, 3^rd^ and 7^th^ post injury days (Figure 1A). FJ-B staining on non-injured and treated with TNF (Group-NA) and treated with PBS (Group-NB) did not show any FJ-B stained neurons at all-time points of the study (Photographs and data are not shown). Quantification of FJ-B stained degenerating neurons showed significant decrease in the number of degenerating neurons in injured mice treated with TNF compared to injured mice treated with PBS at all post injury days studied (1^st^ Post injury day: Student's t-test, p < 0.0001, Welch's approximate t = 8.538 with 6 degrees of freedom; 3^rd^ post injury day: Student's t-test, p = 0.0003, Welch's approximate t = 6.102 with 8 degrees of freedom; and 7^th^ post injury day: Student's t-test, p < 0.0001, Welch's approximate t = 9.839 with 7 degrees of freedom; Figure 1B). There was progressive decrease in the number of degenerating neurons from 1^st^ day to 3^rd^ day (p < 0.01) and 3^rd^ day to 7^th^ day (p < 0.01, Figure 1B).

Immunostaining of sections from injured brain with anti-NeuN antibody for neurons showed denser distribution of neurons around the injury site in injured mice treated with TNF compared to injured mice treated with PBS at 3^rd^ and 7^th^ post injury days (Figure 2A). Immunostaining of sections from non-injured brain treated with TNF (Group-NA) and treated with PBS (Group-NB) with anti-NeuN antibody did not show any difference at all-time points studied (Photograph and data are not shown). Quantification of the NeuN positive neurons around the injury site showed significantly more number of neurons in injured mice treated with TNF compared to injured mice treated with PBS at 3^rd^ and 7^th^ post injury day (Student's t-test, 3^rd^ day: p < 0.001, Welch's approximate t = 4.71 with 8 degrees of freedom; 7^th^ day: p < 0.0001, Welch's approximate t = 7.307 with 10 degrees of freedom, Figure 2B) indicating decreased neurodegeneration in mice treated with TNF. Quantification and comparison of neurons in non-injured brain treated with TNF (Group-NA) compared to non-injured mice treated with PBS (Group-NB) at all-time points studied did not show any difference (Data not shown). In injured and treated with TNF (IA group) number of neurons progressively increased from 1^st^ day to 3^rd^ day (p < 0.05) and 3^rd^ day to 7^th^ day (p < 0.05), but not in injured and treated with PBS (IB group).

### 3.2. Effect of TNF on astrocytes around the injury site

**Figure 3.**
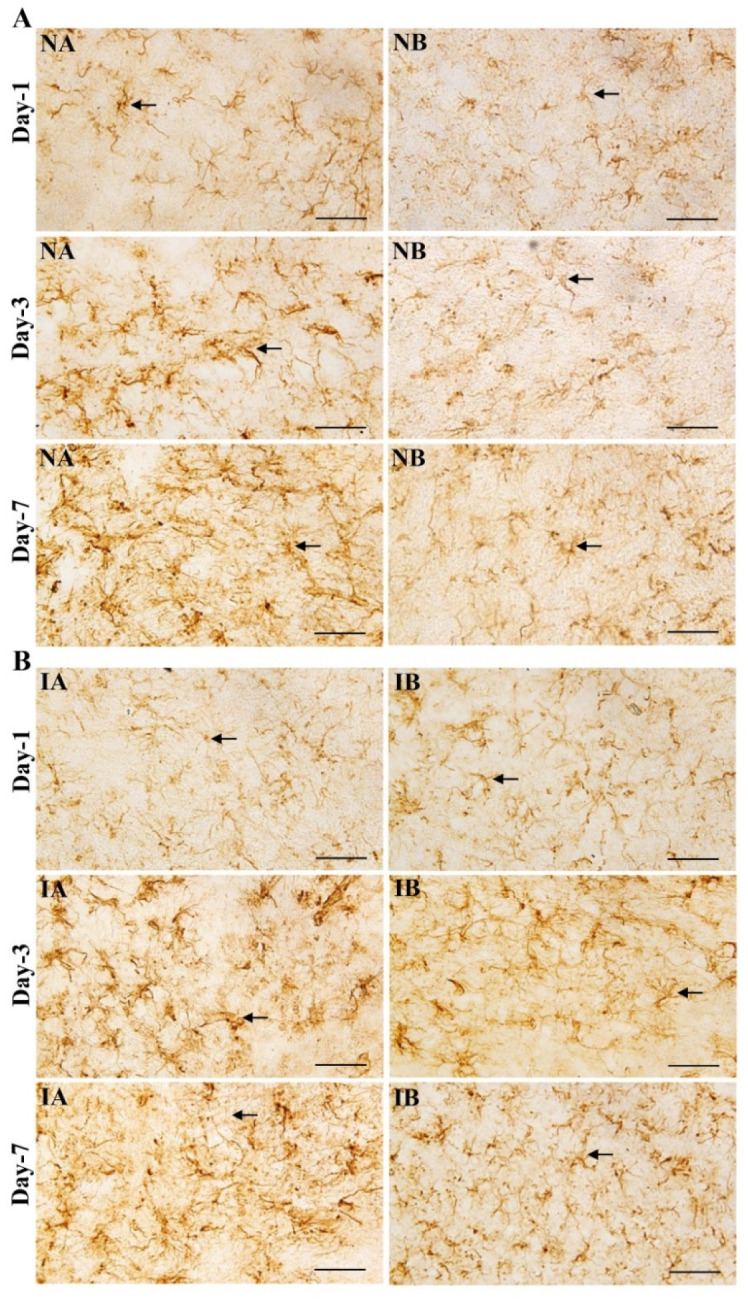
(A) Photomicrographs showing glial fibrillary acidic protein (GFAP) immunostaining (arrows) in the brain section of non-injured mice treated with TNF (NA) and mice treated with PBS (IB) at 1^st^, 3^rd^ and 7^th^ day. Note the increase in the distribution of astrocytes in non-injured and treated with TNF (NA) mice at 3^rd^ and 7^th^ day compared to non-injured and treated with PBS (IB). Scale bar = 50 µm. (B) Photomicrographs showing glial fibrillary acidic protein (GFAP) immunostaining (arrows) in the brain section of injured mice treated with TNF (IA) and injured mice treated with PBS (IB) at 1^st^, 3^rd^ and 7^th^ day. Note the increase in the density of astrocytes distribution in injured and treated with TNF (IA) mice at 3^rd^ and 7^th^ day compared to injured and treated with PBS (IB). Scale bar = 50 µm.

**Figure 4.**
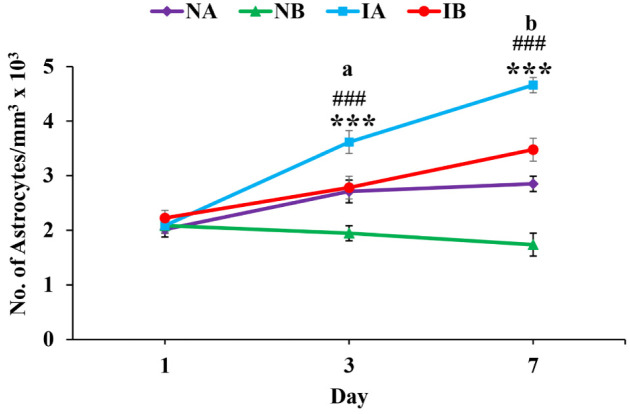
Graph showing the number of astrocytes at 1^st^, 3^rd^ and 7^th^ days in stab wound injured mice treated with TNF (IA, n = 6 for each time point) or treated PBS (IB, n = 6 for each time point) and age matched non-injured mice treated with TNF (NA, n = 6 for each time point) or treated with PBS (NB, n = 6 for each time point). Note significantly increased number of astrocytes in injured mice treated with TNF (IA) compared injured mice treated with PBS (IB) at 3^rd^ (One-way ANOVA, Bonferroni multiple comparison test, IA vs IB, *** p < 0.001) and 7^th^ (IA vs IB, *** p < 0.001) day. Similarly, note significantly increased number of astrocytes in non-injured mice, treated with TNF (NA) compared non-injured mice treated with PBS (NB) at 3^rd^ day (NA vs NB, ^###^ p < 0.001) and 7^th^ day (NA vs NB, ^###^ p < 0.001). Further also note that in both IA and IB number of astrocytes progressively increased from 1^st^ day to 3^rd^ day (^a^ p < 0.05) and 3^rd^ day to 7^th^ day (^b^ p < 0.05). However, such a progressive increase in the number of astrocytes is not seen in non-injured groups treated with TNF or PBS (NA and NB).

Brain sections immunostained for GFAP, showed an increase in the distribution of astrocytes in non-injured mice treated with TNF to a smaller extent and in injured mice treated with TNF-α treated to a greater extent on 3^rd^ and 7^th^ day compared to non-injured and injured mice treated with PBS (Figure 3A and 3B). Quantification of the number of astrocytes around the injury site showed a significant increase in both non-injured and injured treated with TNF at 3^rd^ and 7^th^ day compared to respective PBS treated mice (Figure 4, One-way ANOVA, Bonferroni multiple comparison test—3^rd^ day: IA vs IB, p < 0.001, NA vs NB, p < 0.001, F = 74.52, df = 3,20; 7^th^ day: IA vs IB, p < 0.001, NA vs NB, p < 0.001, F = 283.92, df = 3,20). In both non-injured brain and injured brain treated with TNF, increase in the astrocytes was significant and progressive from 1^st^ day to 3^rd^ day (p < 0.05) and 3^rd^ day to 7^th^ day (p < 0.05) (Figure 4). However, such a progressive increase in the number of astrocytes is not seen in non-injured groups treated with TNF or PBS (NA and NB).

**Figure 5.**
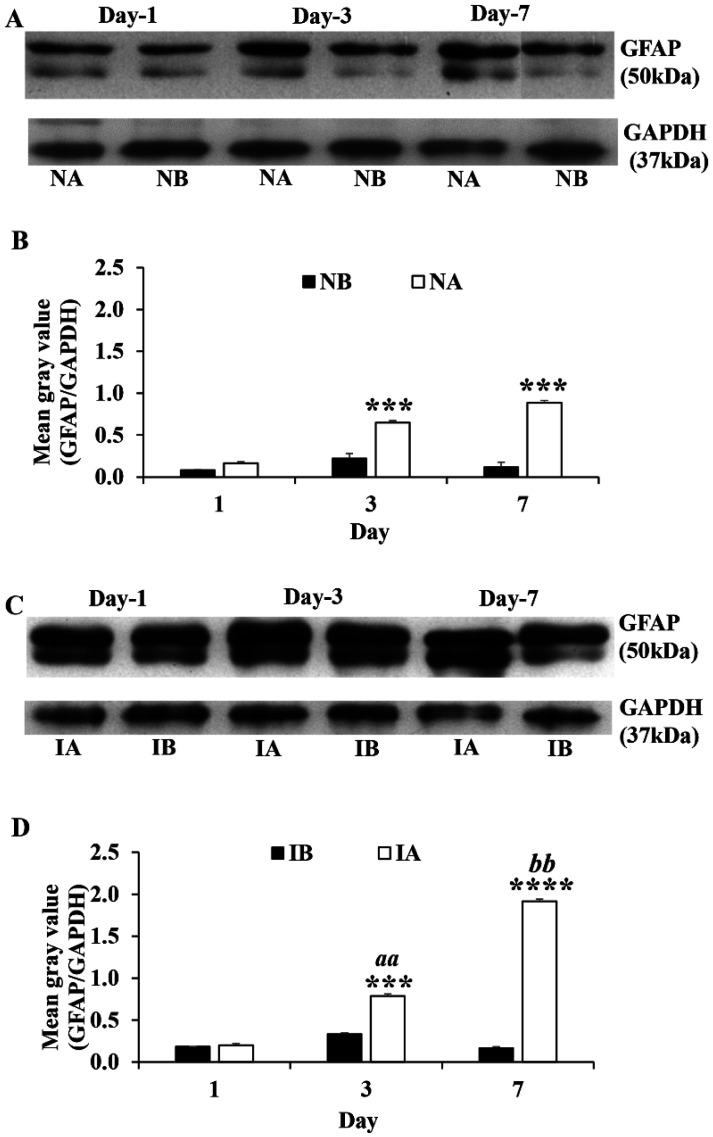
(A) Glial fibrillary acidic protein (GFAP) immunoblotting from 1^st^, 3^rd^ and 7^th^ day tissues from non-injured mice treated with TNF (NA, n = 6 for each time point) and non-injured mice treated with PBS (NB, n = 6 for each time point) probed with anti-GFAP and anti-GAPDH antibodies. (B) Graph showing the mean grey value (GFAP/GAPDH) of the immunoblots. Note significantly increased GFAP content in NA compared to NB at 3^rd^ (Student's t-test, *** p < 0.001) and 7^th^ days (*** p < 0.001). (C) Glial fibrillary acidic protein (GFAP) immunoblotting from 1^st^, 3^rd^ and 7^th^ day tissues from injured mice treated with TNF (IA, n = 6 for each time point) and injured mice treated with PBS (IB, n = 6 for each time point) probed with anti-GFAP and anti-GAPDH antibodies. (D) Graph showing the mean grey value (GFAP/GAPDH) of the immunoblots. Note significantly increased GFAP content in IA compared to IB at 3^rd^ day (*** p < 0.001) and 7^th^ day (**** p < 0.0001). Further note that GFAP contents significantly increased from 1^st^ day to 3^rd^ day (^aa^ p < 0.01) and 3^rd^ day to 7^th^ day (^bb^ p < 0.01) in injured mice treated with TNF. However, such a progressive increase in the GFAP content is not seen in non-injured groups treated with TNF (NA) or PBS (NB).

Western blot analysis for GFAP showed significantly enhanced GFAP content in both non-injured (Figure 5A, B) and injured mice (Figure 5C, D) treated with TNF compared to PBS-treated group on 3^rd^ and 7^th^ post injury days [Student's t-test, Non-injured groups (3^rd^ day: NA vs NB, p < 0.001, t = 11.25, df = 6; 7^th^ day: NA vs NB, p < 0.0001, t = 31.11, df = 6, Figure 5A, B); Injured groups (3^rd^ day: IA vs IB, p < 0.001, t = 28.39, df = 6; 7^th^ day: NA vs NB, p < 0.0001, t = 25.65, df = 6, Figure 5C, D)]. Further the GFAP contents significantly increased from 1^st^ day to 3^rd^ day (p < 0.01) and 3^rd^ day to 7^th^ day (p < 0.01) in injured mice treated with TNF (3^rd^ day vs 7^th^ day, p < 0.001). However, such a progressive increase in the GFAP content was not seen in non-injured groups treated with TNF (NA) or PBS (NB).

**Figure 6.**
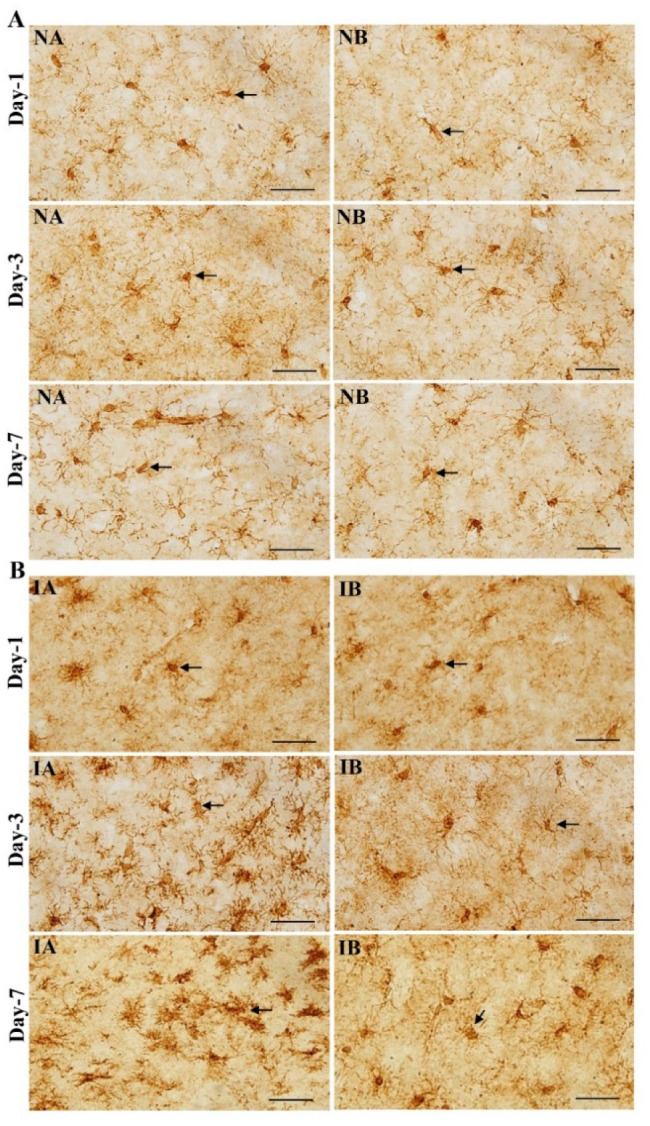
(A) Photomicrographs showing ionized calcium binding adaptor molecule-1 (Iba1) immunostaining in brain sections from non-injured mice treated with TNF (NA) and non-injured mice treated with PBS (NB) at 1^st^, 3^rd^ and 7^th^ day. Note the increase in the microglia distribution in non-injured mice treated with TNF (NA) compared to non-injured mice treated with PBS (NB) at 3^rd^ and 7^th^ day. Scale bar = 50 µm. (B) Photomicrographs showing ionized calcium binding adaptor molecule-1 (Iba1) immunostaining in brain sections from injured mice treated with TNF (IA) and injured mice treated with PBS (IB) at 1^st^, 3^rd^ and 7^th^ day. Note the increase in the microglia distribution in injured mice treated with TNF (IA) compared to injured mice treated with PBS (IB) at 1^st^, 3^rd^ and 7^th^ day. Scale bar = 50 µm.

### 3.3. Effect of TNF on microglia around the injury site

**Figure 7.**
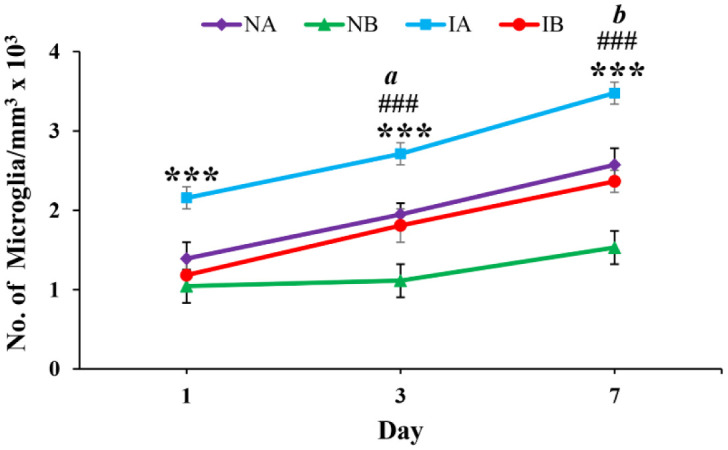
Graph showing the number of microglia at 1^st^, 3^rd^ and 7^th^ days in stab wound injured mice treated with TNF (IA, n = 6 for each time point), stab wound injured mice treated PBS (IB, n = 6 for each time point), age matched non-injured mice treated with TNF (NA, n = 6 for each time point) and age matched non-injured mice treated with PBS (NB, n = 6 for each time point). Note significantly increased number of microglia in injured mice treated with TNF (IA) compared to injured mice treated with PBS (IB) at 1^st^ day (One-way ANOVA, Bonferroni multiple comparison test, IA vs IB, *** p < 0.001), 3^rd^ day (IA vs IB, *** p < 0.001) and 7^th^ day (IA vs IB, *** p < 0.001). Similarly, note significantly increased number of microglia in non-injured mice treated with TNF (NA) compared non-injured mice treated with PBS (NB) at 3^rd^ day (NA vs NB, ^###^ p < 0.001) and 7^th^ day (NA vs NB, ^###^ p < 0.001). Further, note that number of microglia significantly increased from 1^st^ day to 3^rd^ day (^a^ p < 0.05) and 3^rd^ day to 7^th^ day (^b^ p < 0.05) in injured mouse treated with TNF. However, such a progressive increase in the microglia is not seen in non-injured groups treated with TNF (NA) or PBS (NB).

Brain sections immunostained with Iba-1 showed increased density microglial distribution in both non-injured and injured mice treated with TNF on 1^st^, 3^rd^ and 7^th^ day (Figure 6A and 6B). Quantification of the microglia around the injury site showed significant increase in the number of microglia in both non-injured and injured mice treated with TNF compared to PBS treated mice (Figure 7) (One-way ANOVA, Bonferroni multiple comparison test: 1^st^ day: IA vs IB, p < 0.001, F = 37.35, df = 3,20; 3^rd^ day: IA vs IB, p < 0.001, NA vs NB, p < 0.001, F = 42.07, df = 3,20; 7^th^ day: IA vs IB, p < 0.001, NA vs NB, p < 0.001, F = 164.54, df = 3,20; Figure 7). Further, the number of microglia significantly increased from 1^st^ day to 3^rd^ day (^a^ p < 0.05) and 3^rd^ day to 7^th^ day (^b^ p < 0.05) in injured mice treated with TNF and PBS. However, such a progressive increase in the microglia is not seen in non-injured groups treated with TNF (NA) or PBS (NB).

### 3.4. Effect of TNF on expression of BDNF around the injury site

**Figure 8.**
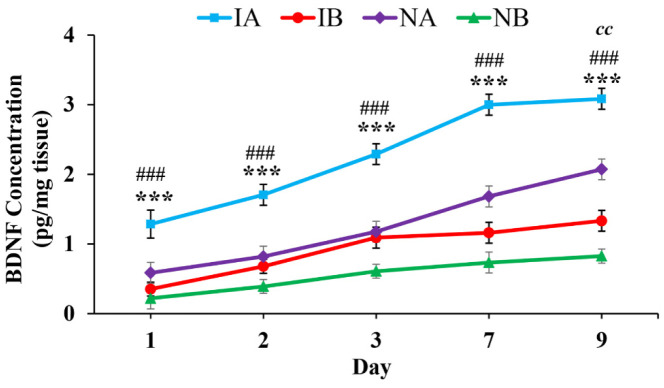
Graph showing the concentration of BDNF on 1^st^, 2^nd^ , 3^rd^ , 7^th^ and 9^th^ day in the tissue around the injury site in the stab wound injured mice treated with TNF (IA, n = 6 for each time point), stab wound injured mice treated with PBS (IB, n = 6 for each time point), non-injured mice treated with TNF (NA, n = 6 for each time point), and non-injured treated with PBS (NB, n = 6 for each time point). Note significantly increased BDNF content in injured mice treated with TNF (IA) compared injured mice treated with PBS (IB) at all-time points (One-way ANOVA, Bonferroni multiple comparison test, IA vs IB, *** p < 0.001). Similarly, note significantly increased BDNF content in non-injured mice treated with TNF (NA) compared non-injured mice treated with PBS (NB) at all-time points (One-way ANOVA, Bonferroni multiple comparison test, NA vs NB, ^###^ p < 0.001). Further note that BDNF content significantly increased from 1^st^ day to 9^th^ day (1^st^ day vs 9^th^ day: ^cc^ p < 0.01) in injured mouse treated with both TNF and PBS. However, such a progressive increase in the BDNF content is not seen in non-injured groups treated with TNF (NA) or PBS (NB).

BDNF level was significantly increased in non-injured and injured mice treated with TNF compared to mice treated with PBS at all-time points studied (Figure 8; One-way ANOVA, Bonferroni multiple comparison test: 1^st^ day: IA vs IB, p < 0.001, NA vs NB, p < 0.001, F = 910.67, df = 3,20; 2^nd^ day: IA vs IB, p < 0.001, NA vs NB, p < 0.001, F = 809.17, df = 3,20; 3^rd^ day: IA vs IB, p < 0.001, NA vs NB, p < 0.001, F = 1262.1, df = 3,20; 7^th^ day: IA vs IB, p < 0.001, NA vs NB, p < 0.001, F = 2424.7, df = 3,20; 9^th^ day: IA vs IB, p < 0.001, NA vs NB, p < 0.001, F = 2943.9, df = 3,20). Increase in the BDNF concentration in the tissue around the injury site was progressive from day-1 to day-9. The BDNF content significantly increased from 1st day to 9^th^ day (1^st^ day vs 9^th^ day: p < 0.01) in injured mouse treated with both TNF and PBS. However, such a progressive increase in the GFAP content is not seen in non-injured groups treated with TNF (NA) or PBS (NB).

## 4. Discussion

In this study, we have demonstrated activation of the astrocytes (astroglyosis) and microglia (microgliosis) in brains of both normal mice (non-injury) and stab wound injured mice treated with TNF for 1, 3 or 7 days. We observed decreased neurodegeneration (alternatively neuroprotection) in stab wound injured mice that are treated with TNF for 3 or 7 days compared to injured mice that are treated with vehicle. Neurodegeneration decreased progressively from 1^st^ day to 3^rd^ day and 3^rd^ day to 7^th^ day in stab-wound injured and treated with TNF as well as in those treated with PBS. Further the BDNF content in the tissue around the injury site in stab-wound injured + TNF treated (treated for 1, 2, 3, 7 and 9 days) mice was significantly increased. The observed neuroprotection may be related to glio-stimulatory role of extraneously infused TNF, where in TNF stimulated both astrocytes and microglia to become activated. These activated glial cells might have released neurotrophic factor BDNF, which in-turn protected the neurons from degeneration in the stab-wound injury. Our results are consistent with a previous study with similar conclusions [48] and the role of other cytokines IL-1β and IFN-γ in astrogliosis, microgliosis and neuronal protections in stab wound injury [49].

### 4.1. TNF neurotoxicity in stab wound neurodegeneration

We observed degenerating neurons, especially on the first day, in and around the injury site in injured mice treated with both PBS and TNF. This initial neurodegeneration may be due to several toxic substances released from the injured cells and may be due to toxicity of TNF itself (from infused and/or intrinsic TNF). It is proposed that TNF not only has neuroprotective role, it also plays a negative role in the nervous system. It has been shown that high level of TNF is associated with the progression of a variety of neurological disorders, including Alzheimer's disease, as well as Parkinson's disease [50],[51].

Neurodegeneration also may be due to localized activation of neuroinflammation by TNF, which could initiate neuronal death, even though TNF has not been demonstrated to cause neuronal death in healthy brain [52]. In an animal model of cerebral ischemia, a dramatic up-regulation of TNF protein and mRNA has been shown [53],[54]. TNF expression has been shown to increase preceding the onset of neuronal cell death [41]. In an in-vitro model of neurodegenerative disease, it was confirmed that pro-inflammatory cytokines interleukin-1β (IL-1β) and TNF are elevated in the neurodegenerative disease state and induced neuronal death and apoptosis [55].

The observed neurodegeneration in our study also may be through activation of microglia, indeed our results showed increased microglia in the injured brain. Although it is assumed that microglia are the major source of TNF during neuroinflammation [56], even astrocytes and neurons produce TNF [57]. During neuroinflammation, different inflammatory stimuli activate microglia and activate several signaling pathways (p38MAPK, NF-κB) [58],[59]. TNF de novo production and release during the neuroinflammatory process, is mediated by infiltrated CD4+ and CD8+ T cells. These cells release the IFN-γ which, via the MEK/ERK signaling pathway, induce microglia for increased de novo TNF production and release.

The mechanism of TNF neurotoxicity is claimed to be through release of excessive glutamate. TNF can potentiate cytotoxicity by (i) inhibiting glutamate transport on astrocytes and by rapidly triggering the expression AMPA and NMDA receptors, (ii) decreasing inhibitory GABA_A_ receptors on neurons [26]. Thus, excessive TNF harms the central nervous system and the harmful effect is through release of excessive glutamate. It is well known that when extracellular glutamate accumulates, it inhibits synaptic activity or kills the neurons and associated synapses as well [60].

Neurodegeneration observed in our study may be mediated through TNF's TNFR1 receptor which activates the caspase family, which induce cell death. Involvement of TNFR1 in neurodegeneration in stab-wound injury needs to be explored in future studies. TNF acts through its two receptors TNFR1 and TNFR2. Both receptors are expressed in various tissues, with TNFR2 being more specific to the cells of the immune system [61]. TNFR1 is ubiquitously expressed on nearly all cells. TNFR2 is restricted to T lymphocytes and other cells [62]. TNFR2 is expressed by cells of myeloid lineage, peripheral T cells and alveolar lymphocytes and macrophages [63]. TNFR2 is limited in both its expression and its activation. Expression of TNFR2 is restricted to cell types, including endothelial cells, fibroblasts and subsets of neurons and immune cells (myeloid cells, T- and B-cell subsets) [64],[65].

### 4.2. Role of TNF in neuroprotection in stab wound injury

Our results showed a neuroprotection by TNF in the stab-wound injury. It may be through neurotrophic factors released from activated astrocytes and/or microglial cells. Alternatively, it may be direct beneficial effect of TNF as it is claimed to be neuroprotective also. TNF is a pro-inflammatory cytokine that regulates several biological processes, including apoptosis, differentiation, proliferation, as well as activation of neurons [66]. TNF regulates inflammation when released at very low levels [67]. TNF has also been linked with resolution of inflammation [4]. Peripherally derived TNF can exert its effect through the HPA axis and the vagus nerve to stimulate various CNS responses, such as neuroendocrine responses, sleep and fever, and immune system regulation [68]. Peripheral infused TNF can act directly on the brain by entering the CNS through the blood brain barrier (BBB) either by active transport mechanisms or passive diffusion [68],[69]. This proves the crucial role of TNF in normal brain physiology. Above studies are in support of our hypothesis that, extraneously infused TNF may act on the nervous system. In healthy CNS, TNF is released at very low levels by a variety of brain cells including neurons [67]. TNF, in addition to having neuroprotective role, shows an important role in synaptic plasticity [23],[24]. Recent studies indicate that there is a neuroprotective and function-maintaining role for TNF, and TNF is essential in maintaining synaptic plasticity, as well as learning and memory formation [70]. In a study conducted on mice, it was found that mice with absent TNF or deleted TNF-receptors displayed significantly poor learning and impaired cognitive function, which shows that low levels of TNF under non-inflammatory immune conditions is important for normal cognitive function [71].

### 4.3. Role of TNF in Astrogliosis in stab-wound injury

Our results showed astrogliosis in both normal and injured mice treated with TNF. X. Hu et al. showed enhancement of GFAP in the normal Muller's glial cell to a smaller extent and to a larger extent in preactivated Muller glial cells by treatment with TNF [39]. Marked increase in the levels of TNF and also transcripts for interleukin (IL)-1a, IL-1b was reported in a nitrocellulose membrane stab injury model in the adult mouse. This elevation of TNF was associated with the increase in glial fibrillary acidic protein mRNA, an early manifestation of astrogliosis, an observation consistent with our study [72],[73]. Astrocytes play major role in neuroinflammation. They are the regulators of immune responses (innate and adaptive) in the injured neural tissue. Astrocyte activity may enhance the inflammatory reactions and tissue damage or facilitate immunosuppression and tissue repair depending on timing and context [74]. Experiments show that the loss of gliosis during the early phases of injury results in exacerbation and persistence of inflammatory cells. Astrocytes depletion during the chronic phase of experimental autoimmune encephalomyelitis (EAE) ameliorates disease and reduces leukocyte infiltration into the CNS [75],[76].

Several other studies also indicate a strong association between TNF and astrocytes in in neural injury/lesion models, which are comparable to our stab-wound injury models. Exposure of astrocytes in tri-culture medium (medium formulated to support neurons, astrocytes, and microglia) to LPS (Lipopolysaccharides) hypertrophies the astrocytes, increases the secretion of a number of pro-inflammatory cytokines (e.g. TNF, IL-1α, IL-1β, and IL-6). Following mechanical trauma in the tri-culture showed increased astrocyte migration towards the site of injury. Microglia in the tri-culture plays a significant neuroprotective role during glutamate-induced excitotoxicity [77]. Above literature are in agreement with our argument that extraneously infused TNF, along with intrinsic TNF released from cells in and around injury site stimulate astroglial cells to become activated, which in-turn play their role in neuroprotection. Significant increase in number of astrocytes and GFAP content at 3^rd^ and 7^th^ in stab wound injured and treated with TNF compared with PBS treated groups suggest the gliosis under the influence of infused TNF. The TNF stimulatory effect is sustained as there was a significant increase from 3^rd^ day to 7^th^ day in both groups treated with TNF and PBS. This may be due to combined action of intrinsic and infused TNF. The enhancement of astrocytes to a smaller extent in injured and treated with PBS may be due to action of intrinsic TNF released from the activated astrocytes, microglia or both.

### 4.4. Role of TNF in Microgliosis in stab-wound injury

Microglia exhibit both beneficial and detrimental effects in neuroinflammation. Results of our study showed enhanced number of microglial cells in the injured brain treated with TNF at 3^rd^ and 7^th^ post injury days compared to that injured and treated with PBS. Activated microglia produce protective factors that help to prevent neuronal injury, such as BDNF, glial cell-derived neurotrophic factor (GDNF) and nerve growth factor (NGF) [77],[78]. In our study we have shown enhancement of BDNF in TNF treated group, which is consistent with the earlier reports [79],[80]. However, we have not studied other neurotrophic factors. There is a need for analyzing the other neurotrophic factors and their role in stab-wound injury.

Microglia, when activated stimulate an adaptive immune response. They release several inflammatory mediators like proinflammatory cytokines, chemokines, prostaglandins, inducible nitric oxide synthase (iNOS), cyclooxygenase-2 (COX-2), free radicals [81]. Purpose of such reactions are for the repair and the restoration of the homeostasis. However, it may also lead to complications, resulting in tissue injury [82]. In our study we have not addressed the question whether such inflammatory mediators are released from the activated microglial cells and astrocytes. It is likely that, activated microglial cells release such mediators as reported by others, and tend to aggravate the inflammation. However, it appears that even though such mediators are released they are not at high levels, as we observed neuroprotection rather than neurodegeneration. Further studies on these inflammatory mediators in stab-wound injury is required.

Activation of microglial receptors (P2X4Rs) leads to increased levels of BDNF [79]. Activated microglia also produce pro-inflammatory cytokines which tend to increase the inflammation [80]. Microglia release large amounts of TNF during inflammation, an important component of the neuroinflammatory reaction. Microglia have shown an important role in mechanisms of neuropathic pain following neurodegeneration through purinergic signaling, especially through expression of P2X and P2Y receptors (P2XR and P2YR) [83]. Activation of microglial P2X4Rs shown to increase the levels of BDNF [83]. BDNF reported to alter transmembrane anion gradient in subpopulations of lamina-1 neurons in the spinal cord dorsal horn by downregulating the neuronal chloride transporter KCC2, contributing to neuropathic pain [84].

Above literature on microglia agree with our argument that extraneously infused TNF, along with intrinsic TNF released from cells in and around injury site stimulate microglial cells to become activated, which in turn play their role in neuroprotection. Significant increase in number of microglia at 1^st^, 3^rd^ and 7^th^ day in stab wound injured and treated with TNF compared with PBS treated groups suggest the gliosis under the influence of infused TNF. The TNF stimulatory effect on microglia appears to be sustained as there was a significant increase from 1^st^ day to 3^rd^ day and 3^rd^ day to 7^th^ day in both groups treated with TNF and PBS. This may be due to combined action of intrinsic and infused TNF. The enhancement of microglia to a smaller extent in injured and treated with PBS may be due to action of intrinsic TNF released from the activated astrocytes, microglia or both.

### 4.5. Role of BDNF in neuronal survival in stab wound injury

Our results showed a positive correlation between increased BDNF release and enhanced survival of neurons in mice that are injured and treated with TNF. Studies indicate the physiological relationship between BDNF and astrocytes [85]. Studies on injured adult spinal cord showed that BDNF increased dramatically compared to normal, non-injured spinal cord, and it was associated with enhanced neuron survival and axon regeneration, an observation consistent with our study. Binding of BDNF into its receptor TrkB, activates several pathways and induces the expression of genes related to neuronal proliferation, survival, and inflammatory response [86]. Studies have shown that binding of BDNF into its other receptor, p75NTR, increases apoptotic and inflammatory signaling in neurons and glial cells [75],[76]. Recent studies suggest that BDNF may also be a potential target for new treatment strategies. In animal models of neurodegenerative diseases, therapy with BDNF in vivo and in vitro showed promising results [87]. Neurogenesis and regional neuronal activity increased after BDNF infusion into the hippocampus of adult rats [88]. Previous studies indicate the physiological relationship between BDNF and astrocytes [80]. In addition, other studies on injured adult spinal cord showed that BDNF increased dramatically compared to normal, non-injured spinal cord, and it was associated with enhanced neuron survival and axon regeneration [89].

As discussed above, in our study activated astrocytes and microglia after treatment with TNF may be responsible for increased release of BDNF as well as neuronal protection. However, we have not addressed the question on the source of BDNF, weather it is from activated astrocyte or activated microglia or both. Further studies are required to address this question.

Significant increase in BDNF contents at all-time points studied in stab wound injured and treated with TNF compared with PBS treated groups may be the key factor in neuroprotection. The TNF's sustained stimulatory effect on microglia and astrocytes might have caused sustained and a significant increase in BDNF from 1^st^ day to 9^th^ day in both groups treated with TNF and PBS. This may be due to combined action of intrinsic and infused TNF. The enhancement of BDNF to a smaller extent in injured and treated with PBS may be due to action of intrinsic TNF released from the activated astrocytes, microglia.

Thus, TNF has both neuroprotective and neurodegenerative effects. TNF is produced both in the normal tissue and injured tissue. The neuroinflammatory process is associated with activation of microglia, astrocytes and release of the TNF and BDNF. Being a pro-inflammatory cytokine, TNF when it is in excess, aggravates further gliosis and inflammation. When TNF is at its moderate level, it facilitates resolving of inflammation and hence neuroprotection. The BDNF, released from activated microglia and astrocytes counteract aggravation of inflammation induced by excessive TNF. It is hard to determine whether the observed effects (gliosis and neuroprotection), in our study are from the extraneous infused TNF or both intrinsic and extrinsic source. It may be the combined effect of both intrinsic and extrinsic source. The receptor through which it mediates its effects needs to be studied. Similarly, BDNF may be from activated astrocytes, microglia or both, which needs to be determined in future studies.

## 5. Conclusions

This study indicates that TNF activates astrocytes (astroglyosis) and microglia in normal brain (non-injury) and stab wound injury. TNF protects the cortical neurons and decreases their degeneration in stab wound injury. This neuroprotection may be through enhancement of BDNF. Enhancement of BDNF probably by either by activated astrocytes or activated microglia or both. Further experiments are needed to find weather activated astrocyte or microglia or both play their role in enhancement of BDNF or any other mechanisms exist. Results of our study is promising to use TNF as therapeutic agent to treat neural injuries and neurodegenerative diseases, however several additional experiments are needed to validate a few things such as to know the different dose effects of TNF, adverse effects of extraneously infused TNF, to find the source of BDNF (weather from astrocyte or microglia). This study supports strategies of preserving the activating astrocytes and microglia in the planning of future therapies to promote regeneration in brain injury.
